# Structure and Dynamics of the Misfolding Intermediate in the Pathogenic T183A Prion Protein Mutant

**DOI:** 10.1021/acs.jctc.5c00742

**Published:** 2025-10-04

**Authors:** Carmen Biancaniello, Alessandro Emendato, Alfonso De Simone

**Affiliations:** Department of Pharmacy, 9307 University of Naples Federico II, Via D. Montesano 49, Naples 80131, Italy

## Abstract

Characterizing high-energy conformations in protein molecules is crucial to delineate the nature of dynamic processes that underlie biological activity, as these elusive species often play critical roles in fundamental mechanisms of the cell, such as enzyme catalysis or protein folding and aggregation, among many others. In this context, the integration of molecular simulations with experimental biophysics represents a powerful strategy to delineate complex conformational landscapes, enabling the study of transient conformations at atomic resolution. Here, we characterized intermediate states along the misfolding pathway of the human prion protein (PrP) variant T183A, which is associated with familial Creutzfeldt–Jakob disease. Using replica-averaged molecular dynamics simulations, restrained with nuclear magnetic resonance chemical shifts, we obtained structural ensembles showing enhanced conformational heterogeneity for the T183A variant compared with the WT protein. The mutant ensemble was found to populate partially misfolded states characterized by disruption of the β-sheet and local unfolding of key helical regions of the protein. Additionally, dynamic cross-correlation analyses evidenced significant loss of cooperative fluctuations across secondary structure elements, delineating how structural destabilization in the T183A variant leads to the insurgence of misfolding intermediates. Collectively, these findings provide critical insights into the underlying mechanisms of T183A-induced PrP misfolding and its consequent aggregation into amyloid fibrils.

## Graphical abstract

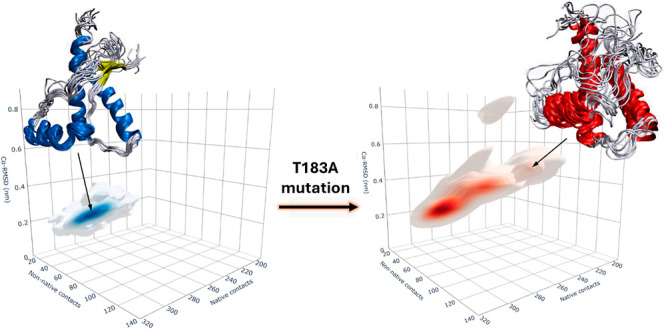

## Introduction

It has long been recognized that structural fluctuations play critical roles for the biological activity of macromolecules. Backbone and side chain dynamics in protein molecules drive key processes such as enzymatic catalysis, protein–protein interactions, or cellular signaling.^1–5^ The biological role of these fluctuations is, however, extremely challenging to delineate for current experimental techniques, which are primarily tailored to study well-defined and rigid protein states.^6^ This challenge is critical when characterizing the structure of intermediate species in off-equilibrium mechanisms, including protein misfolding and aggregation into amyloid fibrils.^7–9^ These processes are of critical relevance, as they are inherently connected with the underlying mechanisms of aberrant neurodegenerative disorders such as Alzheimer’s and Parkinson’s diseases.^10^ In this context, the major challenge in addressing the origins of protein misfolding stems from the study of intermediate species on the pathway of amyloid formation, including monomers,^11,12^ oligomers,^13,14^ and protofibrils,^15,16^ as their transient nature and reduced population make them difficult to isolate and study at high resolution.

To characterize the structural properties of a misfolding intermediate, we here employed experimentally restrained replica-averaged molecular dynamics (RAMD)^17^ simulations incorporating chemical shifts (CSs) data^18,19^ from nuclear magnetic resonance (NMR). In this approach, multiple independent copies (replicas) of the same system are simulated simultaneously, and NMR parameters, back-calculated from each replica throughout the simulations, are used as ensemble-averaged restraints.^20,21^ With this approach, we elucidated the conformational properties of high energy conformational intermediates promoting aberrant aggregation of a pathological mutant of the C-terminal domain of the prion protein (PrP). The misfolding of the cellular PrP (PrP^C^) into a scrapie fibrillar form (PrP^Sc^) is indeed associated with transmissible spongiform encephalopathies (TSE), also known as prion diseases.^22^ PrP is a highly conserved monomeric glycoprotein primarily expressed in neuronal cells, where it is located on the outer plasma membrane via a glycosylphosphatidylinositol (GPI) anchor.^23^ Its biological function remains debated, with putative functions ranging from long-term potentiation to copper sensing, long-term memory, and calcium regulation.^24–26^ In the pathological context, in addition to sporadic and infective forms of TSEs, which are observed in ∼85% of the cases, mutations of the PrP gene (PRNP) have been found in association with inherited forms of prion diseases such as familial Creutzfeldt–Jakob disease (fCJD), fatal familial insomnia, and Gerstmann–Sträussler–Scheinker disease (GSS).^27–29^ Currently more than 50 TSE pathological mutants of PrP are known,^30^ most of which involve single amino acid substitutions in the C-terminal globular domain (residues 125–230), with a smaller number observed in the disordered N-terminal domain (residues 23–124) of the protein. Among these mutations, T183A, has been associated with early onset fCJD and dementia.^31^ This pathological mutation has been shown to strongly destabilize the PrP^C^ fold^32^ through the loss of a hydrogen bond between the backbone amide of Y162 and the side chain of T183 (Figure S1), leading to a reduction of more than 25 °C in the thermal stability of the C-terminal domain of PrP^C^ (huPrP^C^_125–230_).^11^ Our structural refinement, incorporating experimental CS via the artificial neural network-based model NapShift,^18^ aimed at characterizing the detailed native-state free energy of T183A huPrP^C^_125–230_ to identify possible aggregation-prone intermediates. We found that the mutation significantly enhances the dynamical content of the globular domain of huPrP^C^ by boosting both local and global motions of the protein. The loss of correlated motions between secondary structure elements of the protein was found to promote low-populated partially misfolded states, thereby revealing the structural pathway of the early stages of T183A huPrP^C^_125–230_ misfolding. The thermal accessibility to these misfolded states provides a mechanistic explanation for the lack of lag-phase in the aggregation kinetics of this mutant PrP construct.

## Materials and Methods

### CS-Restrained MD Simulations

CS-restrained MD simulations were carried out as averages across multiple replicas using a modified version of the GROMACS software package^33^ that integrates NapShift,^18^ an artificial neural network-based model trained to calculate backbone atoms CSs (δ^15^N, δ^13^C, δ^13^Cα, δ^13^Cβ, δ^1^Hα, and δ^1^HN) from structure. NapShift enables the integration of experimental CS data as restraints in biomolecular simulations, as it can be derivatized in the Cartesian space. The implementation of NapShift has been shown to enhance the conformational sampling toward accurate energy landscapes to generate ensembles that optimally represent the experimental CSs.^18,21^ Using NapShift at each simulation step to predict CSs from the protein conformation, an energy penalty is computed as a harmonic potential based on the difference between the predicted and reference experimental CS values, as given by eq 1

1 $$V^{CS}=K\sum_{i}^{N_{res}}\sum_{j}^{6}{\left(\delta_{ij}^{experimental}-\delta_{ij}^{back‐calculated}\right)}^{2}$$

where *V* ^CS^ is the hybrid restraint potential energy, δ_*ij*_^experimental^ is the experimental CS, δ_*ij*_^back‑calculated^ is the NapShift-predicted CS, *i* and *j* represent the residue number and backbone atom type, respectively, and *K* > 0 is the weight of the restraint with respect to the empirical force field. This penalty is expressed as a differentiable function of the atomic coordinates, enabling the calculation of the forces to minimize the deviation between the predicted and experimental CSs. As a result, this experimentally driven energy term is added to the standard force field, giving the total potential energy *V* ^Total^ (eq 2) used in our calculations, where *V* ^FF^ is the potential energy of the underlying force field.

2 $$V^{Total}=V^{FF}+V^{CS}$$

The CS restraints of NapShift were applied as flat-bottom potentials, ensuring that no restraining forces were imposed when the predictions fell within the error limits of the experimental values. This setting mitigates the impact of the restraint when minor deviations occur, ensuring that the system’s dynamics are not overly constrained by the experimental data.

### Simulations Setup

The starting structures for the replica simulations were generated from the initial 20 ns simulations coupled with a restraining force *K* of 600 J mol^–1^ ppm^–2^. The NMR structure with PDB code 1HJM^34^ of the huPrP^C^_125–230_ was used to represent the wild-type (WT) protein and to model the T183A variant, since no experimental structures are available for this mutation. Experimental CSs for the native states to use as restraints were obtained from the Biological Magnetic Resonance Bank (BMRB, https://bmrb.io) entries 50527 (WT) and 50528 (T183A).^35^ Simulations were carried out using the Amber ff14SB force field^36^ with the TIP3P water model.^37^ The proteins were solvated in cubic water boxes containing 12,101 TIP3P water molecules and neutralized with three sodium ions. After energy minimization using the steepest descent method, the systems were equilibrated at 300 K and 1 atm in the *NVT* and *NPT* ensembles, respectively. During equilibration, temperature was coupled using the *V*-rescale thermostat^38^ with a coupling constant of 0.1 ps, and pressure was controlled using the Berendsen barostat^39^ with a compressibility value of 4.5 × 10^–5^ bar^–1^. The productive MD simulations were run in the *NPT* ensemble with periodic boundary conditions and a time step of 2 fs. The LINCS algorithm^40^ was used to constrain bonds involving hydrogen atoms, while electrostatic interactions were calculated using the particle mesh Ewald method^41^ with a cutoff distance of 1.2 nm for both van der Waals and Coulomb interactions. Four configurations were extracted from the last 10 ns of these 20 ns simulations, with snapshots taken every 2 ns. These configurations were then used to initiate the replica-averaged CS restrained MD simulations under the same parameter settings, but adding the iterative modulation of temperature and restraining forces.

We ran four independent replicas for both WT and T183A PrP systems, each consisting of 200 sequential cycles of a 1 ns simulation. The simulation protocol involved temperature annealing performed using the *V*-rescale thermostat, where the temperature was linearly ramped from 300 to 400 K during the first 200 ps, maintained at 400 K until 600 ps, and subsequently reduced back to 300 K by 800 ps. The temperature was kept constant for the remainder of each cycle. Simultaneously, the restraining force *K* was initially set at 200 J mol^–1^ ppm^–2^ and decreased to 20 J mol^–1^ ppm^–2^ over the first 200 ps. The restraining force *K* was kept constant until 600 ps, after which it increased back to 200 J mol^–1^ ppm^–2^ by 800 ps and was held steady until the end of the cycle. This setup facilitated the initial unfolding of the prion proteins at high temperature and reduced restraint influence, followed by refolding to the native state under normal temperature and stronger restraints to allow the sampling of intermediate conformations within the basin of the native state.

### Data Collection and Analysis

Data collection included cycles from 21 to 200 by excluding the first 20 cycles during which the system is equilibrating. The conformations of the sampling were extracted every 10 ps from the last 200 ps of each cycle (under 300 K and maximum restraining force, designated as **sampling phase**), resulting in a total of 15,120 configurations for both WT and T183A PrP systems. The analyses of the samplings included the following structural parameters:

- **Root-mean-square deviation (RMSD)** between the experimental CSs and those predicted for all backbone atoms using SPARTA+^42^ on each sampled configuration.
- **RMSD-free** calculated on a validation set composed of 10% of the experimental CS, randomly selected and excluded from the restraint list during the RAMD production.
- **Root-mean-square fluctuation (RMSF) of Cα atoms** by aligning the ensembles on the starting experimental structure (PDB code 1HJM). Structural alignment was based on the Cα atoms of the secondary structured elements.
- **Cα-RMSD** from the native experimental structure, computed on the Cα atoms of residues forming secondary structure elements.
- **Secondary structure assignments** determined using the DSSP program.^43^
- **Native Cα contacts** calculated with an 8 Å cutoff.
- **Dynamical cross-correlation matrix** (DCCM) of all Cα atom pairs by using the R package bio3d,^44^ as implemented by Ichiye and Karplus.^45^ To calculate DCCMs, the Cα atoms of the 3D structure models representing the native conformations of WT and T183A huPrP^C^_125–230_ were selected as the spatial reference frames. MD extracted snapshots were superimposed using the Cα atoms involved in secondary structures only.

Overall, these parameters were selected to capture both local and global structural properties that can help us distinguish between the native state and the different conformations explored by the investigated systems. Calculations were performed using Gromacs analysis routines or in-house Python scripts, unless otherwise specified. Molecular visualization images were produced using VMD software.^46^

### Relaxation Data from the Analysis of CPMG Experiments in Solution NMR

Solution NMR CPMG spectra were previously measured at the ^1^H frequency of 950 MHz,^11^ at 16 °C and pH 7.0. Measurements were performed in triplicate using a huPrP^C^_125–230_ concentration of 100 μM in 100 mM Na_2_HPO_4_, 10% D_2_O, and employing ν_CPMG_ ranging from 50 to 1200 Hz across 16 data points. These data sets were here processed using Bruker TopSpin software and subsequently analyzed based on Korzhnev and Kay.^47^ The cross-peak intensities, *I*_1_, in spectra recorded for different ν_CPMG_ were converted into *R*_2,eff_ values according to *R*_2,eff_ = −1/*T*·ln­{*I*_1_(ν_CPMG_)/*I*_0_}, where *I*_0_ is the peak intensity when the constant time CPMG element is removed. Uncertainties in *R*_2,eff_ were obtained on the basis of three repeat measurements (in cases where errors in *R*_2,eff_ were less than 2%, errors of 2% were assumed). In order to decouple fast motions from slower conformational exchange, the *R*_2,eff_ values obtained from the analysis of high ν_CPMG_ frequencies were employed as a probe of transverse relaxation of the PrP constructs.

## Results

### Structural Ensembles of WT and T183A huPrP^C^_125–230_

To sample the conformational energy landscape of WT and T183A huPrP^C^_125–230_, we performed RAMD simulations restrained with experimental CS using the NapShift method.^18^ Four replicas, sharing the CS restraints, were run for 200 cycles of simulated annealing (1 ns per replica per cycle) until convergence in the phase space exploration. The latter was evaluated by comparing the distributions of pairwise distances of Cα atoms across two subsets of the ensembles (conformations from cycles 21 to 110—first half—and from cycles 111 to 200—second half) (Figure S2). The analysis showed comparable distributions in the two subsets for both the WT and T183A samplings, thereby indicating converging structural properties in the calculations.

The accuracy of the conformations sampled in the two ensembles was then evaluated using experimental data. Very good agreement was observed when comparing experimental CS with those calculated from the structures of the ensembles using an orthogonal method, SPARTA+,^42^ and resulting in deviations close to the standard errors of this program (Figure S3A,B). In addition, an RMSD-free strategy was employed by randomly selecting 10% of the experimental data to constitute a validation set, which was excluded from the restraint list during the RAMD production phase. Despite not being subjected to restraints, the CS of the validation set, as calculated from the RAMD ensembles, showed a very good agreement with experimental data (Figure S3C,D). The deviations between calculated and experimental CS were also used as an additional check of convergence, with both ensembles showing overlapping distributions in the two subsets of the samplings (Figure S4), indicating a complete degree of convergence with respect to the CS sampling. Additional experimental benchmarking of the huPrP^C^_125–230_ structural ensembles was carried out by calculating the local backbone fluctuations. RMSF of the Cα atoms were computed from the structural ensembles to characterize the extent of backbone fluctuations along the protein sequence (Figure 1A). These results were compared with orthogonal experimental NMR data (Figure 1B,C). In particular, in both WT and T183A structural ensembles, the analysis showed high Cα-RMSF values in loops and unstructured terminal regions of huPrP^C^_125–230_. The dynamic profile of the T183A mutant, however, was found to be generally higher than that of the WT construct. Indeed, higher Cα-RMSF values were observed in various mutant regions, including strands S1 and S2, the helix H1 and in the respective connecting loops, and helixes H2 and H3. We then studied the local dynamics of WT and T183A huPrP^C^_125–230_ using Carr–Purcell–Meiboom–Gill (CPMG) experiments in solution NMR. Transverse relaxation rates (*R*_2,eff_), measured with a refocusing ν_CPMG_ frequency of 50 Hz, showed a generalized enhancement of the dynamic behavior of the T183A mutant compared to the WT protein (Figure 1B). Some resonances in the spectra of the mutant were found to be broadened beyond detection due to enhanced relaxation likely arising from conformational exchange of the region encompassing residues 158–175, which spans β-strand S2 and the flanking loop connecting with α-helix H2 (Figure 1B). In addition, enhanced relaxation was measured for helices H1 and H2 and in the loop connecting strand S1 with helix H1. When analyzing the data acquired at a ν_CPMG_ frequency of 1200 Hz, some residues of T183A displayed reduced *R*_2,eff_ values, reaching similar values as measured in the WT construct. This observation reflects the loss of the *R*_ex_ contribution arising from conformational exchange in some residues of T183A, a phenomenon typically associated with microsecond to submillisecond dynamics (Figure 1C). At a ν_CPMG_ of 1200 Hz, however, some regions of the T183A (strand S1, the S1–H1 loop, and the C-terminal segment of helix H2) retained higher *R*_2,eff_ values than the WT, indicating enhanced dynamics also on faster (nanosecond) time scales. Overall, these relaxation data indicate that the T183A mutation, by depleting a fundamental H-bond, thereby weakening the packing of the β-sheet against helix H3, induces conformational exchange in huPrP^C^_125–230_, in agreement with the results derived from the RAMD structural ensembles.

**Figure 1:**
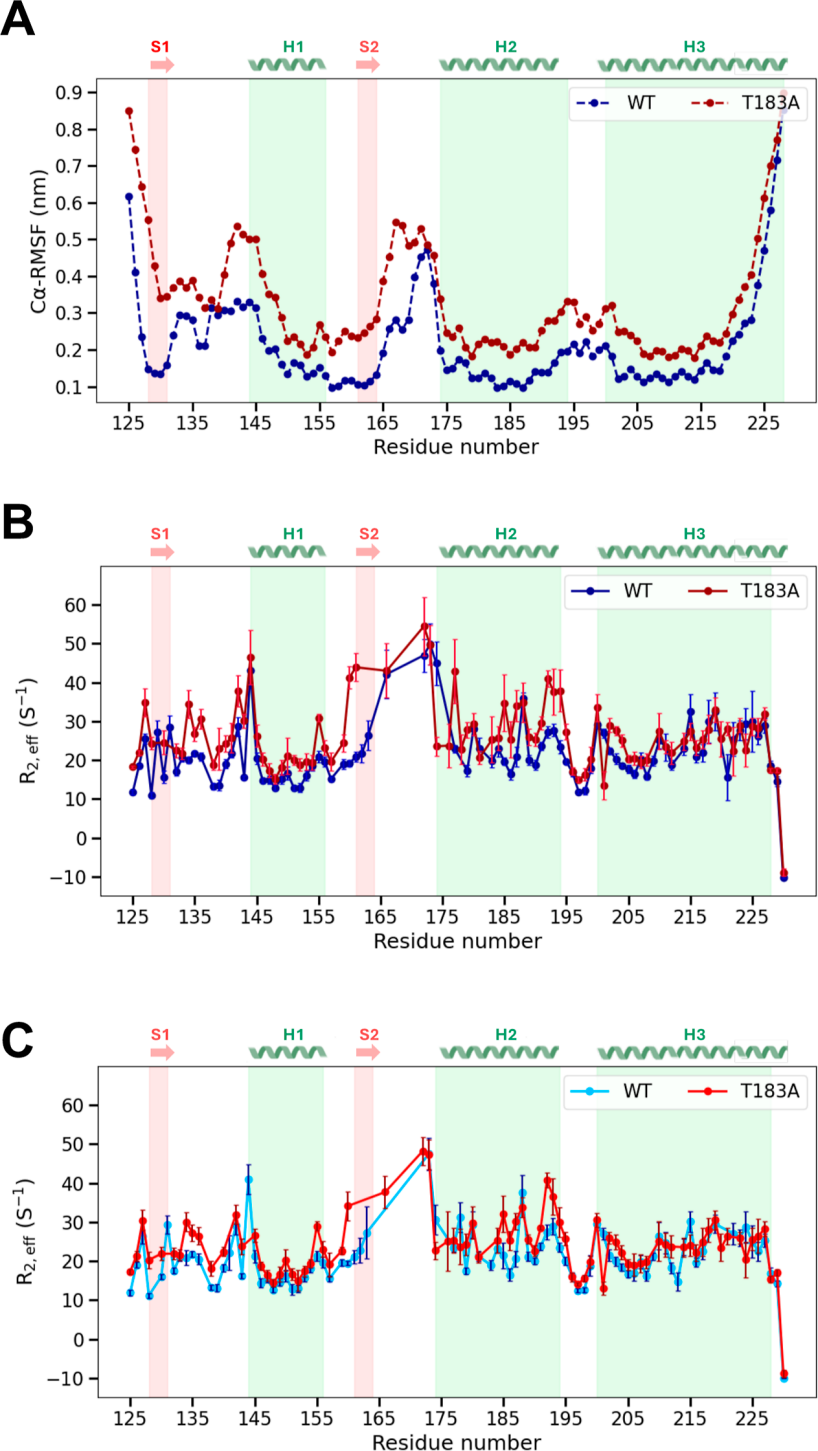
Protein flexibility along the PrP sequence. (A) Cα-RMSF calculated in the structural ensembles of WT (blue) and T183A (red) PrP^C^_125–230_, by aligning the protein structures using Cα atoms of secondary structure elements (highlighted in gray on the plot). (B,C) *R*_2,eff_ measured in NMR CPMG experiments of monomeric, nonaggregated WT (blue), and T183A (red) huPrP^C^_125–230_, at ν_CPMG_ = 50 Hz (B) and ν_CPMG_ = 1200 Hz (C).

### Free Energy Landscapes of WT and T183A huPrP^C^_125–230_

To gain insights into the conformational properties of WT and T183A huPrP^C^_125–230_, we analyzed the structural ensembles generated using CS restraints by projecting the conformations onto three structural variables. These include (i) the number of preserved native contacts (assigned with a cutoff of 8 Å on the Cα–Cα distances), (ii) the number of non-native contacts, and (iii) the Cα-RMSD from the native structure (PDB code: 1HJM). 3D density distribution maps, projected onto these coordinates via kernel density estimation, reflected the conformational free energy landscape of the two structural ensembles (Figures 2 and 3). The maps revealed that the WT construct mostly samples a single dominant cluster, populated by conformations that closely resemble the native structure. These structures mostly preserve the pattern of native contacts and exhibit a low number of non-native contacts and minimal Cα-RMSD from the native structure (Figure 2). In contrast, the T183A sampling showed clear signs of structural heterogeneity (Figure 3). In particular, the conformational distribution of this construct featured four distinct clusters (designated as T183A-1, -2, -3, and -4) characterized by increasing levels of deviations from the native structure (Figure 3). These clusters were identified by detecting the local maxima in the density distribution and assigning each conformation to one cluster based on the proximity in the 3D space. The primary cluster (T183A-1), encompassing 53.6% of the structures, mostly preserves the properties of the native state, sampling conformations relatively similar to those encountered in the single cluster of the WT ensemble. A second conformational cluster, spanning 33.8% of the ensemble structures (T183A-2), shows a partial loss of secondary structure in helix H1 and the N-terminal region of helix H2 (Figure S6, Table 1), resulting in a local reduction of native contacts. In addition to these two native-like clusters, two minor clusters were composed of structures showing significant levels of PrP misfolding, including the T183A-3 (accounting for 10.5% of the conformations), featuring the loss of the β-sheet interface and a partial unfolding of helix H1 (Figure S6). T183A-3 also presented the highest deviations from the native structure when analyzing the distance between Cα atoms of residues 162 and 183 (Figure S7), which describes the packing of the β-sheet onto its native interface with helix H2. This observation is consistent with the loss of stability of strands S1 and S2 in the cluster. The final cluster, T183A-4, accounting for 2% of the conformations, exhibited the highest Cα-RMSD values, relative to those of the native structure. T183A-4 showed significant rearrangement of the packing interface of helix H1 and partial unfolding of both helices H1 and H3 (Figure S6). Overall, the structural features of the clusters identified in the T183A ensemble are consistent with the protection factors measured in NMR H/D exchange experiments.^48^ In particular, these data revealed that some regions of key secondary structure elements of the WT, namely, the N-terminus of H1 and H3 and C-terminus of H2, become deprotected to solvent exchange at acidic pHs. This pattern of deprotected α-helical segments corresponds to unstable secondary structure regions observed in our T183A ensemble (Figure 3), suggesting possible common misfolding pathways between the WT under acidic conditions and T183A at physiological pH.

**Figure 2:**
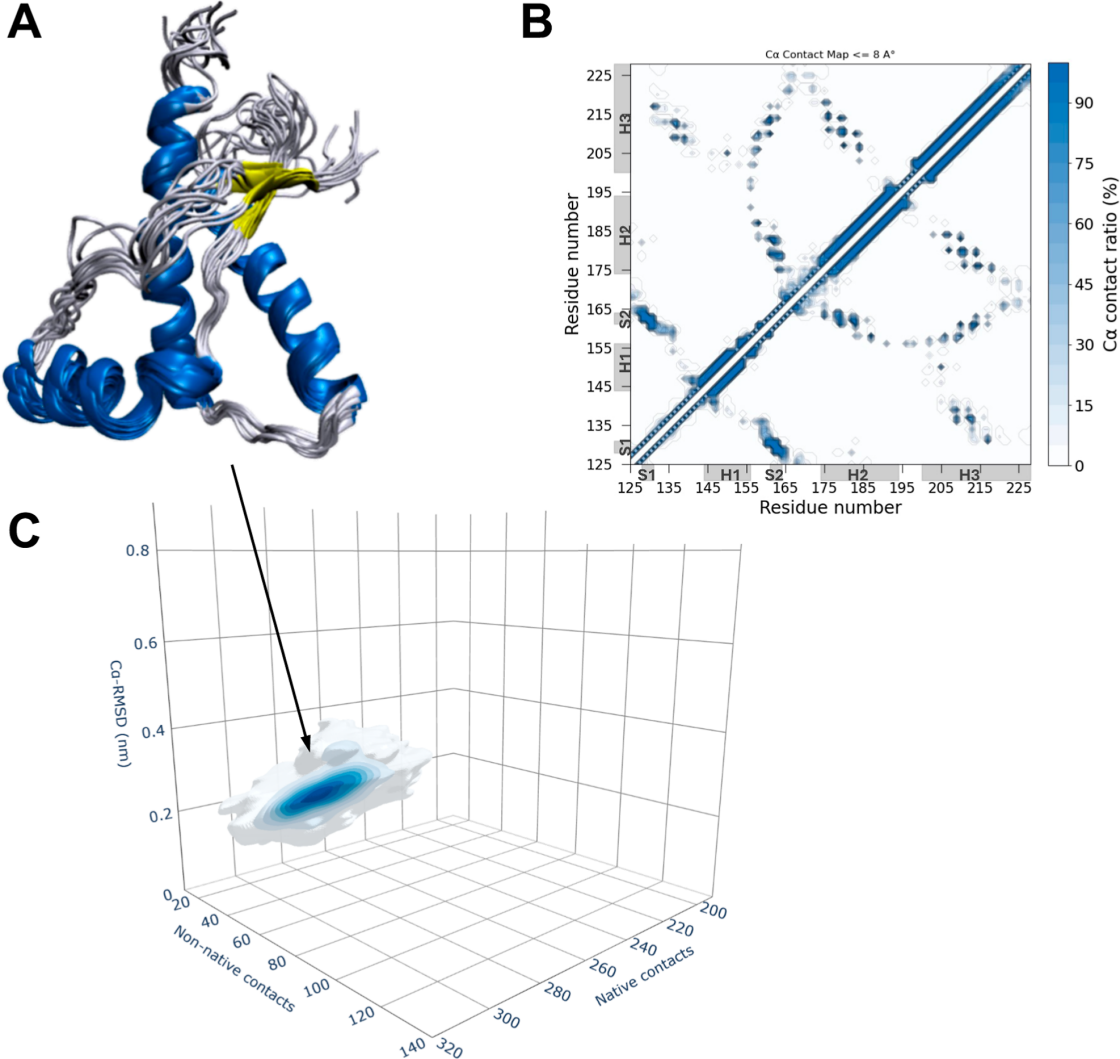
WT huPrP^C^_125–230_ ensemble. (A) A representative structural bundle from the sampling’s conformations is shown by coloring α-helices, β-strands, and coil regions in blue, yellow, and gray, respectively. (B) Contact map averaged onto the conformations of the WT huPrP^C^_125–230_ RAMD ensemble. For each pair of Cα–Cα distances, contacts have been assigned using a cutoff of 8 Å and averaged across the ensemble. The map is represented with a color scale ranging from 0% (white) to 100% (blue). (C) 3D density distribution of the RAMD ensemble projected onto three variables including (i) the number of native Cα contacts (calculated using a cutoff of 8 Å), (ii) the number of new Cα contacts (calculated using a cutoff of 8 Å), and (iii) Cα-RMSD from the native structure (computed on secondary structure regions). The corresponding 2D density maps calculated for each pair variables are reported in Figure S5.

**Figure 3:**
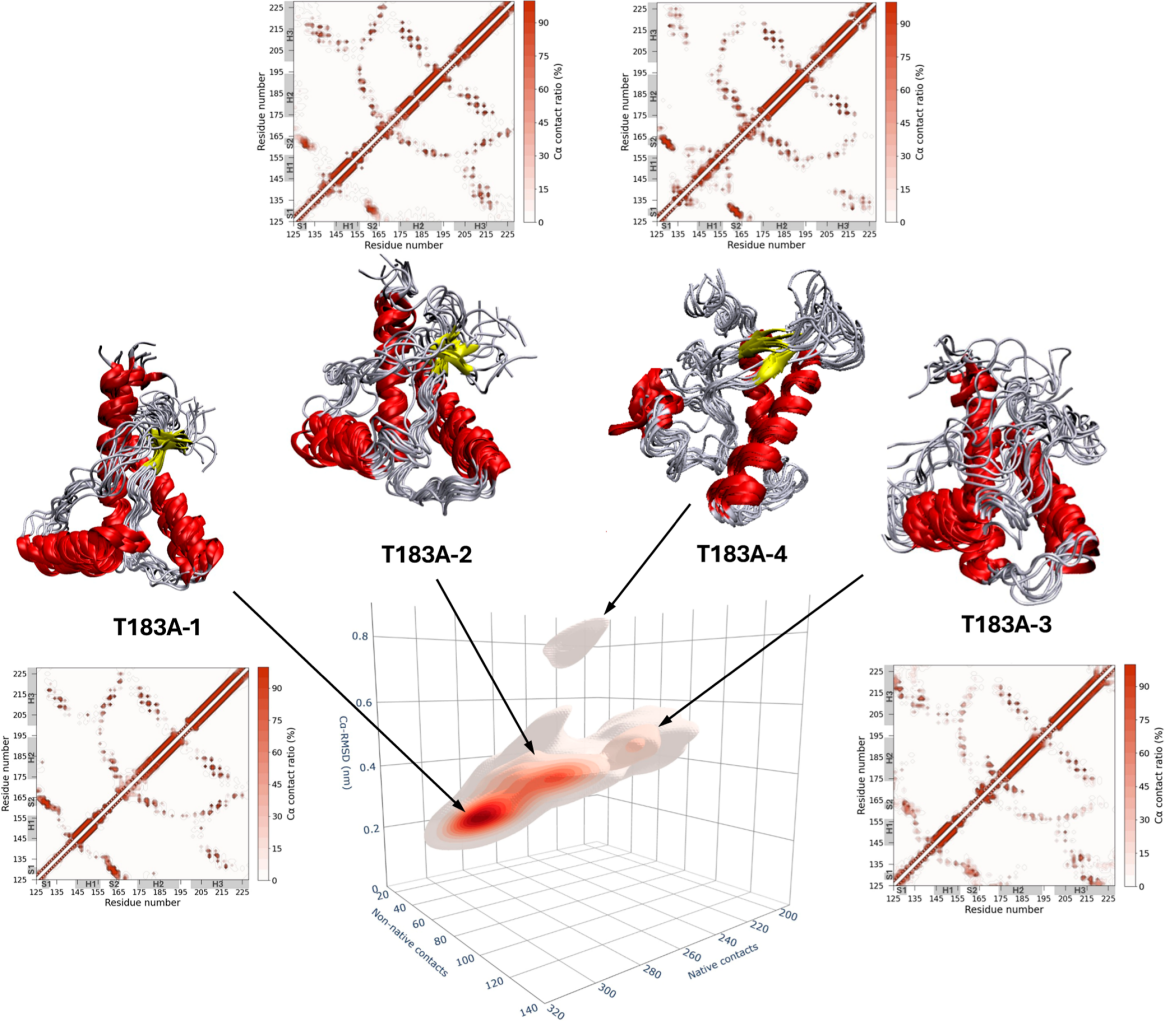
T183A huPrP^C^_125–230_ ensemble. 3D density distribution of the RAMD ensemble projected onto three variables including (i) the number of native Cα contacts (calculated using a cutoff of 8 Å), (ii) the number of new Cα contacts (calculated using a cutoff of 8 Å), and (iii) Cα-RMSD from the native structure (computed on secondary structure regions). The corresponding 2D density maps calculated for each pair variables are reported in Figure S5. The analysis identified four conformational clusters, including one that primarily hosts native conformations (T183A-1) and other three hosting species with increasing levels of structural misfolding (T183A-2, T183A-3, and T183A-4). Values of the clusters’ centroids are reported in Table S1. Representative structural bundles are shown for each cluster by coloring α-helices, β-strands, and coil regions in red, yellow, and gray, respectively. For each cluster, contact maps are calculated by averaging across the cluster conformations, using Cα–Cα distances with a cutoff of 8 Å. The contact maps are represented with a color scale ranging from 0% (white) to 100% (red).

**Table 1:** Total Content of Secondary Structure Elements in Conformational Clusters Identified by RAMD Analysis of WT and T183A huPrP^C^_125–230_ Structures

secondary structure content %	WT 1HJM	WT ensemble	T1831-1	T183A-2	T183A-3	T183A-4
helix	57.3	51.72	52.58	44.9	48.03	41.39
sheet	3.9	5.08	4.54	2.91	0.09	5.44
coil	38.8	43.21	42.88	52.18	51.8	53.17

Collectively, the analysis of the structural ensembles for the two huPrP^C^_125–230_ constructs showed that the wild-type (WT) protein remains stable within a conformational basin that closely resembles that of the native state. In contrast, the T183A variant tends to explore conformations with substantial structural deviations from the native state, with clusters 3 and 4 exhibiting the characteristics of protein misfolding, revealing pathways of local disruption of secondary structural elements and rearrangement of tertiary contacts. The structural information on these conformational transitions is encoded in the experimental CS, as evidenced by the fact that very long MD simulations (5 μs each) predominantly sampled structures very close to the initial conformation for both WT and T183A constructs (Figure S8). In addition, RAMD ensembles generated with a 400 ps sampling phase per cycle (as opposed to 200 ps) produced overlapping 3D distributions of conformations (Figure S9), further supporting the convergence of the simulations.

### Correlated Motions in the PrP Structural Ensembles

In addition to revealing local disruptions in secondary structure elements, the cluster analysis identified large-scale rearrangements in the protein structure, indicating that the T183A mutation enhances long-range fluctuations in huPrP^C^_125–230_. To gain insights into long-range motions in the PrP structure, we calculated the covariance matrix of Cα fluctuations using dynamic cross-correlation matrices (DCCM, Figure 4). These matrices are convenient probes to identify protein regions characterized by a significant degree of correlated or anticorrelated motions.^45^ DCCM revealed considerable differences in the correlation patterns of the WT and T183A huPrP^C^_125–230_ ensembles (Figures 4 and S10). In particular, in the WT ensemble helix H3 (residues 208–217) was found to possess positive correlations (red) with helix H2 (residues 178–184, Figure S10a), with strand S2 (residues 161–163, Figure S10c) and with the regions flanking strand S1 (residues 131, 134, and 135, Figure S10b), as well as anticorrelation with helix H1 (residues 143–152, Figure S10d). Correlated motions were also found between strands S1 and S2 and between the N-terminal tail and the loop region connecting S2 to H2 (Figure S10e). When analyzing the T183A DCCM map, most of the correlations observed in the WT ensemble were absent or significantly reduced. By contrast, new negative cross-correlation peaks were observed between N-terminal regions of H1 and H3 (Figure S10f) and between the N-terminus of H3 (residue 201) and the C-terminus of H2 (residues 186, 189, and 190, Figure S10g). The T183A huPrP^C^_125–230_ ensemble also showed weaker correlations between strand S1 and S2, implying a level of disruption of the antiparallel β-sheet interface (Figure S10h). Further differences were noted for helices H1 and H2, where the terminal regions (residues 143–146 and 155–156 in helix H1, Figure S10i, and residues 192–197 and 181–182 in helix H2, Figure S10j) showed increased anticorrelation in the mutant ensemble. This finding aligns with the structural destabilization and reduction in α-helical content of H1 and H2 shown in the cluster analyses and previous NMR studies.^35,49,50^

**Figure 4:**
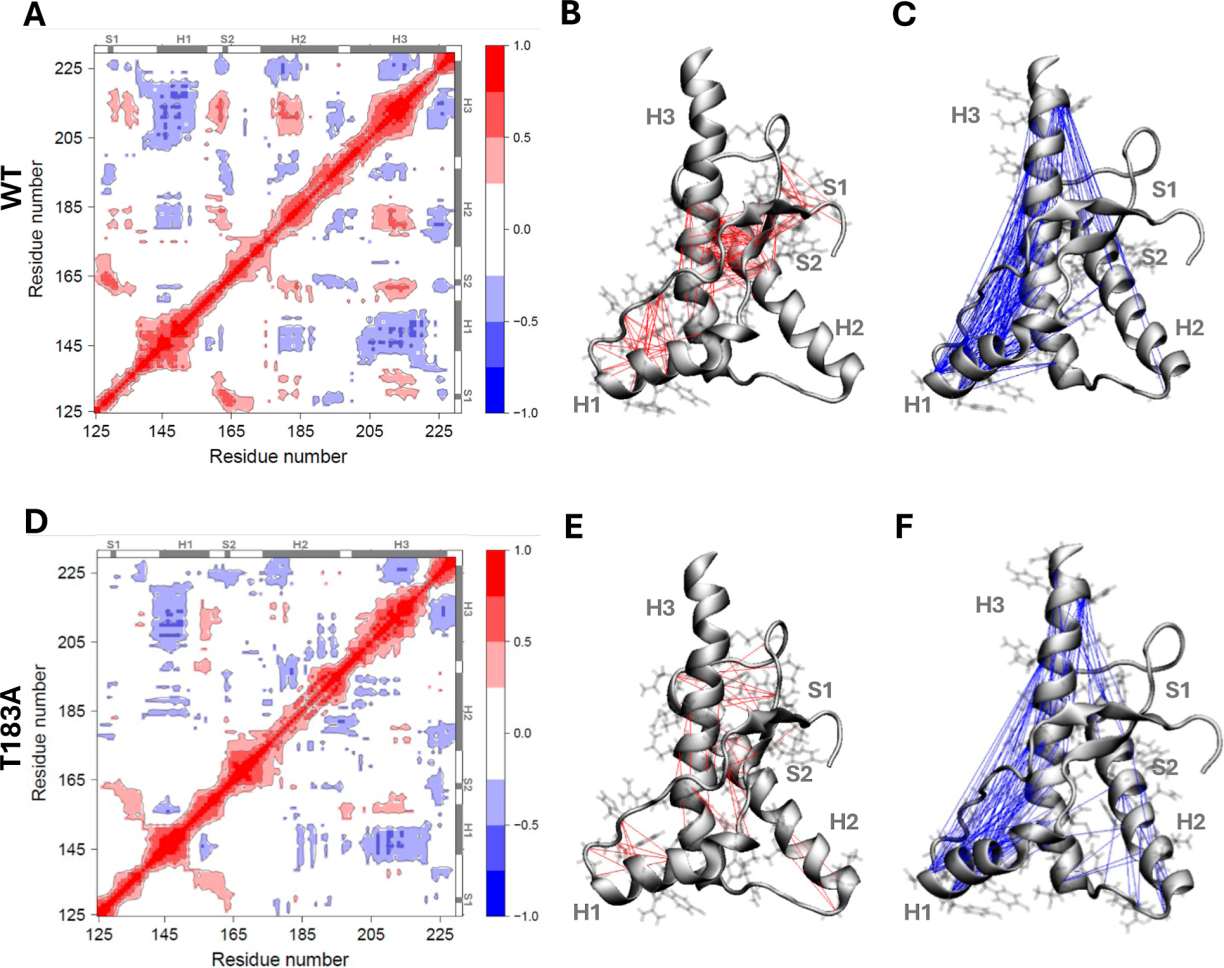
Dynamic cross-correlation matrices in huPrP^C^_125–230_ structural ensembles. For each Cα–Cα atom pair within the ensembles, DCCM are reported using a color-code ranging from +1.0 (red) to −1.0 (blue) and, respectively, indicating highly correlated (red) and anticorrelated (blue) motions between Cα atoms. WT (A) and T183A (D) DCCM are shown with gray bars in the top and right borders, indicating the secondary structure elements in the native state of huPrP^C^_125–230_. (B,E) Correlated motions (correlation coefficients ≥0.4) between Cα–Cα pairs separated by at least of 5 residues in the protein sequence are visualized through red connecting lines on WT (B) and T183A (E) huPrP^C^_125–230_ structure. (C,F) Anticorrelated motions (correlation coefficients ≤ −0.4) between Cα–Cα pairs separated by at least of 5 residues in the protein sequence are visualized through blue connecting lines on WT (C) and T183A (F) huPrP^C^_125–230_ structure.

Taken together, the correlation networks probed by DCCM suggest that the T183A mutation induces several alterations in long-range structural fluctuations of the protein, including the loss of cooperative dynamics between strands S1 and S2 following the partial destabilization of the β-sheet interface. In addition, the mutation reduces the dynamic correlation between helices H2 and H3 and promotes new anticorrelations between the terminal regions of helices H1 and H2. The changes in correlated motions are associated with the sampling of partially misfolded protein clusters such as T183A-3 and T183A-4, respectively, featuring the disruption of the β-sheet and helix H1, including their native-state packing with helices H2 and H3.

## Discussion

The characterization of the conformational transitions inducing the misfolding of PrP is vital for understanding the pathological mechanisms underlying prion diseases and to provide critical structural knowledge that can lead to new potential therapeutic interventions. Developing integrative structural approaches that combine experimental data and simulations is critical to delineate the structural pathways of PrP misfolding at atomic detail, as standard powerful techniques such as X-ray crystallography, NMR, and cryo-EM are unable to capture dynamic intermediate species in these off-equilibrium processes. In this context, RAMD simulations restrained with NMR data represent a powerful approach to achieving a detailed description of heterogeneous protein states at atomic resolution. By accounting of the time and ensemble averaging inherent in NMR experiments, this technique has been effectively combined with various NMR observables, including residual dipolar couplings,^17,51^ H–D exchange,^45^ and relaxation data. Among the NMR observables, CS have proved to be extremely useful structural restraints,^20,52^ as, in addition to be highly accessible and accurate, they provide critical information on both the structural and dynamic properties of protein molecules.

We here applied CS-restrained RAMD to characterize the structures of partially unfolded intermediate species in the PrP variant T183A. This TSE mutation,^32^ which is associated to a familial form of CJD characterized by early onset dementia, has dramatic effects on the stability of the folded domain of PrP. Cellular experiments showed that T183A prevents the native glycosylation at N181 of PrP^C^ and promotes intracellular accumulation of PrP^Sc^ deposits.^53,54^ In addition, the mutation interferes with the folding and maturation of PrP^C^ by disrupting the ligation of the glycosylphosphatidylinositol (GPI) anchor.^55^ NMR studies showed that T183A huPrP^C^ features enhanced dynamics compared to the WT protein, with large-scale motions promoting the population of amyloidogenic intermediates leading to its aggregation into amyloids.^11^ Characterizing the structure of this intermediate is of critical importance to elucidate the mechanism of conversion of T183A huPrP^C^ into huPrP^Sc^.

To characterize the conformational properties of intermediate species in T183A huPrP^C^_125–230_, we exploited the information contained in CS to guide RAMD simulations. Samplings of WT and T183A constructs were carried out until convergence, showing a significant agreement with NMR data. The resulting ensembles revealed that the T183A variant has enhanced structural heterogeneity, as evidenced by higher RMSF values, particularly in strands S1, S2, and their connecting helices, and corroborated by NMR relaxation experiments. In analyzing the free energy landscapes, the WT ensemble was observed to predominantly sample a stable native-like structural cluster, whereas T183A displayed significant conformational heterogeneity across four distinct clusters featuring increasing levels of misfolding. Among these basins, T183A-3 featured the most considerable deviations, including β-sheet disruption and partial unfolding of the helices, reflecting significant protein misfolding.

In terms of global fluctuations, the WT and mutant construct showed considerable differences in correlated motions, with the T183A mutation exhibiting reduced positive correlations with respect to the WT, as well as non-native anticorrelations between the terminal regions of the helices H1 and H2. These conformational changes led to a loss of cooperative dynamics between strands S1 and S2, disrupting the β-sheet interface and reducing dynamic correlation between helices H2 and H3. These data therefore indicate that T183A promotes partially misfolded protein states, such as the T183A-3 and T183A-4 clusters, with notable structural destabilization and native-state packing loss. Together, these findings elucidate the structural implications of the T183A mutation, providing insights into its role in inducing conformational instability and rapid amyloid formation, even by the sole globular domain of PrP^C^ in the absence of the amyloidogenic regions 106–126.^56^

In addition to elucidating the underlying mechanisms of misfolding of PrP, particularly in the context of a TSE mutation, this study flags the importance of combining enhanced conformational sampling with NMR experiments to study heterogeneous protein states of key relevance for biological processes. The introduction of innovative enhanced sampling methods, the definition of new AI-based NMR restraints like NapShift, and the advancements in computational infrastructure will lead to increasingly accurate characterizations of transient high-energy conformational states, significantly enhancing the research focused on identifying therapeutic targets and strategies to combat protein misfolding diseases.

## Supplementary Material



## Notes

Cα–Cα pairwise distances, comparison of calculated and experimental CS, analysis of the simulations convergence, 2D density distributions, secondary structure content in the conformational clusters of WT and T183A samplings, native packing of strand S2 onto helix H2 in the conformational clusters of WT and T183A samplings, conformational distributions of unrestrained ensembles, dynamic cross-correlation matrices, table reporting the values of the collective variables in the conformational clusters, and details, materials, and methods, including photographs of experimental setup (PDF)
