# Supplementary material for: Structure and Dynamics of the Misfolding Intermediate in the Pathogenic T183A Prion Protein Mutant
Source: J Chem Theory Comput. 2025 Oct 4;21(19):9909–18. doi: 10.1021/acs.jctc.5c00742 (PMC12529898; doi:10.1021/acs.jctc.5c00742)
Supplement: Supplementary file 1 [file ct5c00742_si_001.pdf]

Supplementary Material

Structure and Dynamics of the Misfolding Intermediate in the Pathogenic T183A Prion Protein Mutant

Carmen Biancaniello[1], Alessandro Emendato[1] and Alfonso De Simone[1,*]

[1] Department of Pharmacy, University of Naples Federico II, Via D. Montesano 49, Naples 80131, Italy.

*correspondence: alfonso.desimone@unina.it

This PDF file includes:

Table S1
Figures S1 to S10

Table S1. Values of variables in local maxima points identified from the 3D-density distribution analysis of the RAMD WT and T183A PrP ensembles.

CLUSTER	*Cα-RMSD (nm)*	*Native contacts*	*Non-native contacts*
WT Ensemble	0.23	280	37
T1831-1	0.23	283	35
T183A-2	0.37	261	65
T183A-3	0.47	233	87
T183A-4	0.79	249	65

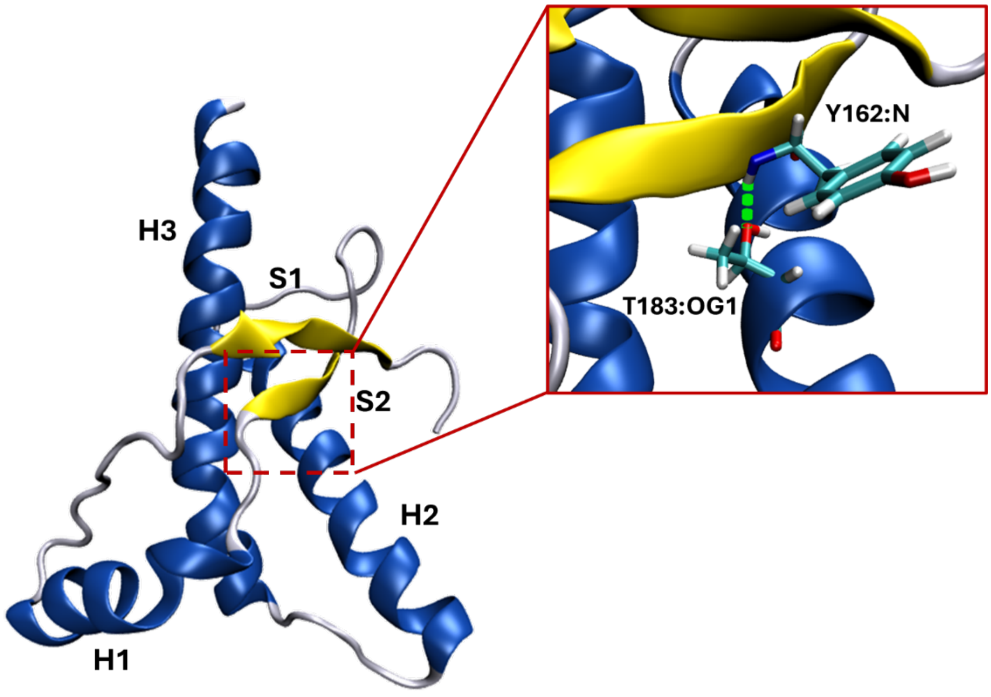

Figure S1. Structure of the C-terminal domain of Human PrP^{C}. huPrP$^{C}_{125-230}$ is composed of three α-helices (H1, residues 144 - 156; H2, residues 174 -194; H3, residues 200 - 230) and a short antiparallel β-sheet comprising S1 (residues 128 - 131) and S2 (residues 161 - 164). The domain is further stabilised by a disulfide bridge between helices H2 and H3 and two N glycosylation sites at residues N181 and N197.[1-4] A key feature is illustrated in the close-up view, highlighting the hydrogen bond between the sidechain of T183 and the backbone amide of Y162, which is depleted as a result of the T183A mutation.

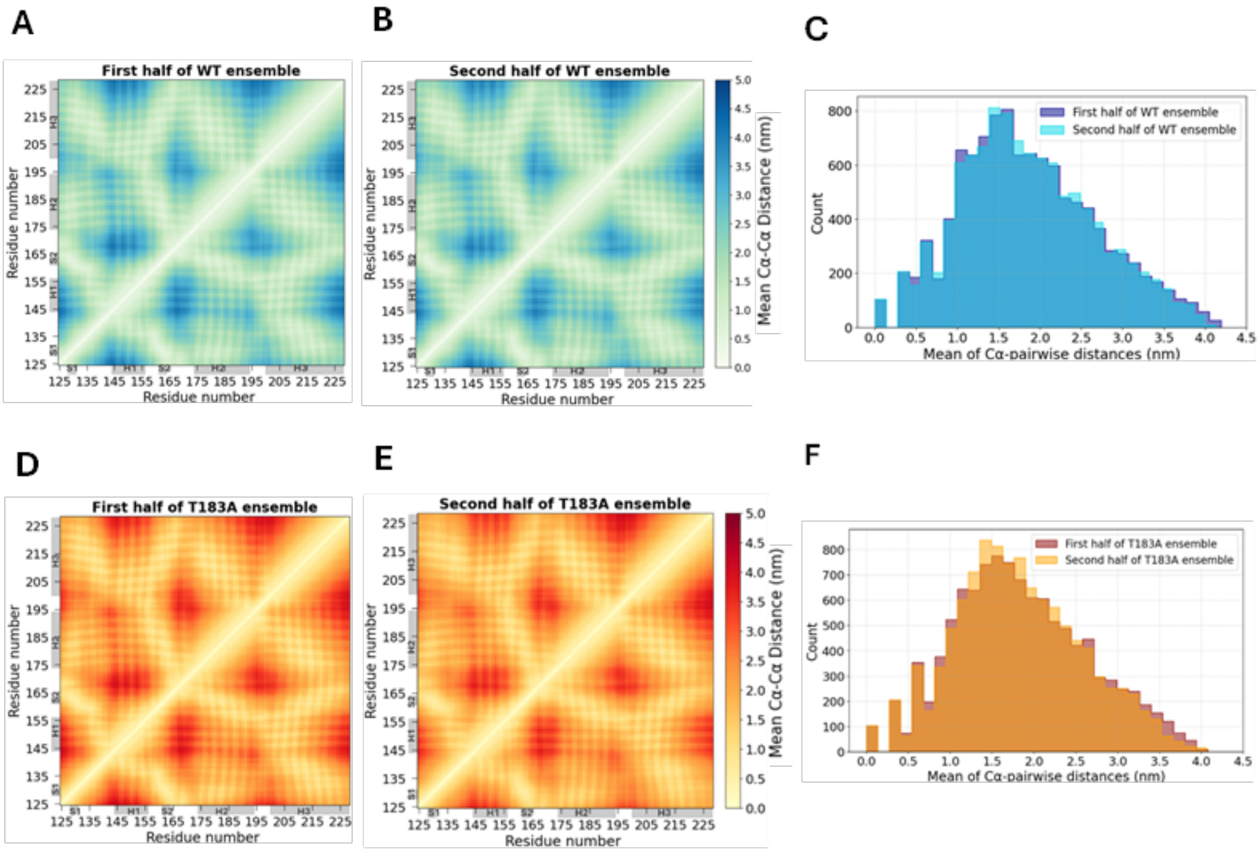

Figure S2. Cα-Cα pairwise distances and samplings convergence. Convergence of NMR-restrained RAMD simulations was assessed using Cα-pairwise distances. Models from each replica of the $huPrP^{C}_{125\text{-}230}$ samplings were divided into cycles 21 to 110 (first half) and cycles 111 to 200 (second half). **(A-B)** Maps of mean Cα-Cα distances for each residue pair in the first half (cycles 21–110) and second half (cycles 111–200) of the WT ensemble. **C)** Distribution of mean Cα-pairwise distances for the first (dark blue) and second (light blue) halves of the WT ensemble. **(D-E)** Maps of mean Cα-Cα distances for the first (cycles 21–110) and second (cycles 111–200) halves of the T183A ensemble. **F)** Distribution of mean Cα-pairwise distances for the first (red) and second (orange) halves of the T183A ensemble.

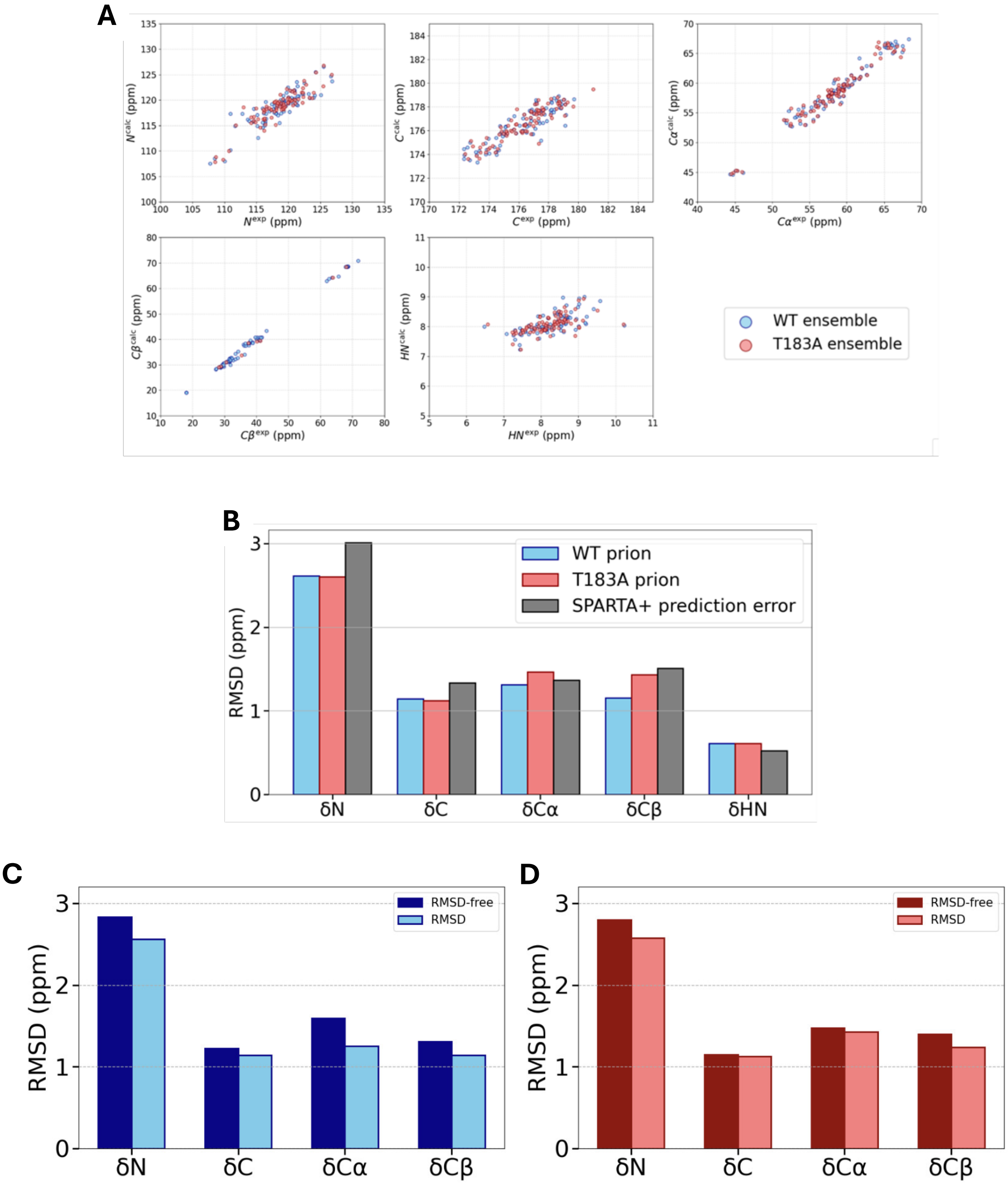

Figure S3. Comparison of calculated and experimental CS. In this analysis, the CS calculation from huPrP$^{C}_{125\text{-}230}$ structures was performed using SPARTA+,[5] which is an orthogonal method to the NapShift[6] restraints employed to produce the samplings. **A)** Scatter plots between experimental and calculated CS of N, C, Cα, Cβ, and HN are shown for the full structural ensembles of WT (blue) and T183A (red). **B)** RMSD values of CS calculated using SPARTA+ from the structural ensembles of WT (blue) and T183A (red). The standard error of SPARTA+ with respect to the 2022 protein CS-structure database used for the definition of NapShift[6] is reported in grey. **C-D)** RMSD-Free values were calculated on a subset comprising 10% of the experimental CS (validation set) from the restraint list during the RAMD production. CS of the validation set were then predicted from the RAMD ensembles using SPARTA[5] and employed to calculate the RMSD-free for the WT (**C**) and T183A (**D**) ensembles.

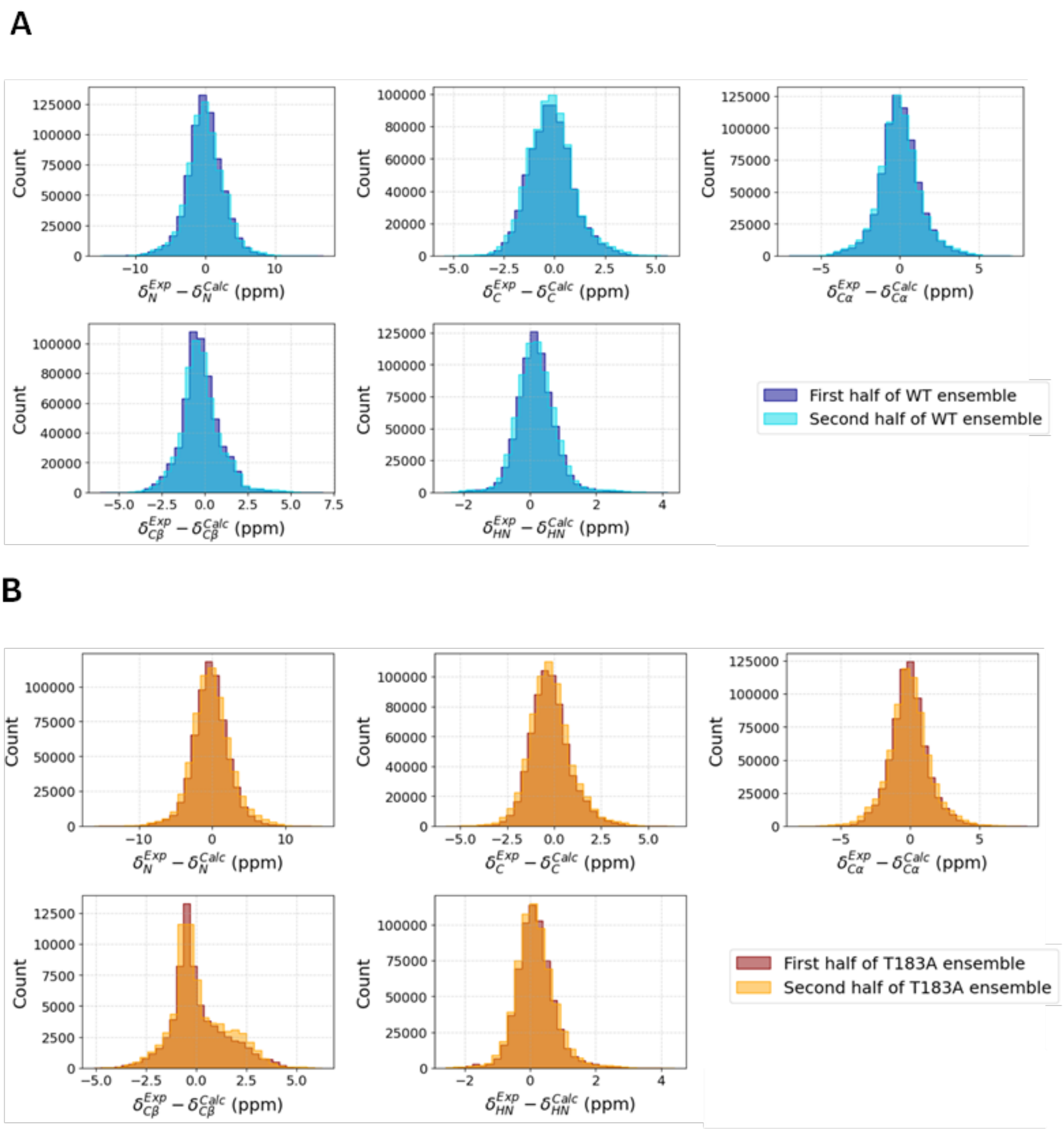

Figure S4. Simulations convergence. Distribution of the differences between experimental and predicted chemical shifts (CS), calculated for N, C, Cα, Cβ, and HN atoms using the structures of the WT **(A)** and T183A **(B)** huPrP$^{C}_{125\text{-}230}$ ensembles. In this analysis, the CS calculation from structures was performed using and SPARTA+,[5] which is an orthogonal method to the NapShift[6] restraints employed to produce the samplings. In order to assess the convergence of the simulations, the samplings were divided into two consecutive segments. Models from each replica of WT PrP were divided into two subsets: cycles 21 to 110 (first half) and cycles 111 to 200 (second half). The resulting distributions across the first and second halves showed highly converging results. **A)** WT ensembles from the first half (dark blue) and second half (light blue) of the simulation cycles. **B)** T183A ensembles from the first half (dark red) and second half (orange) of the simulation cycles.

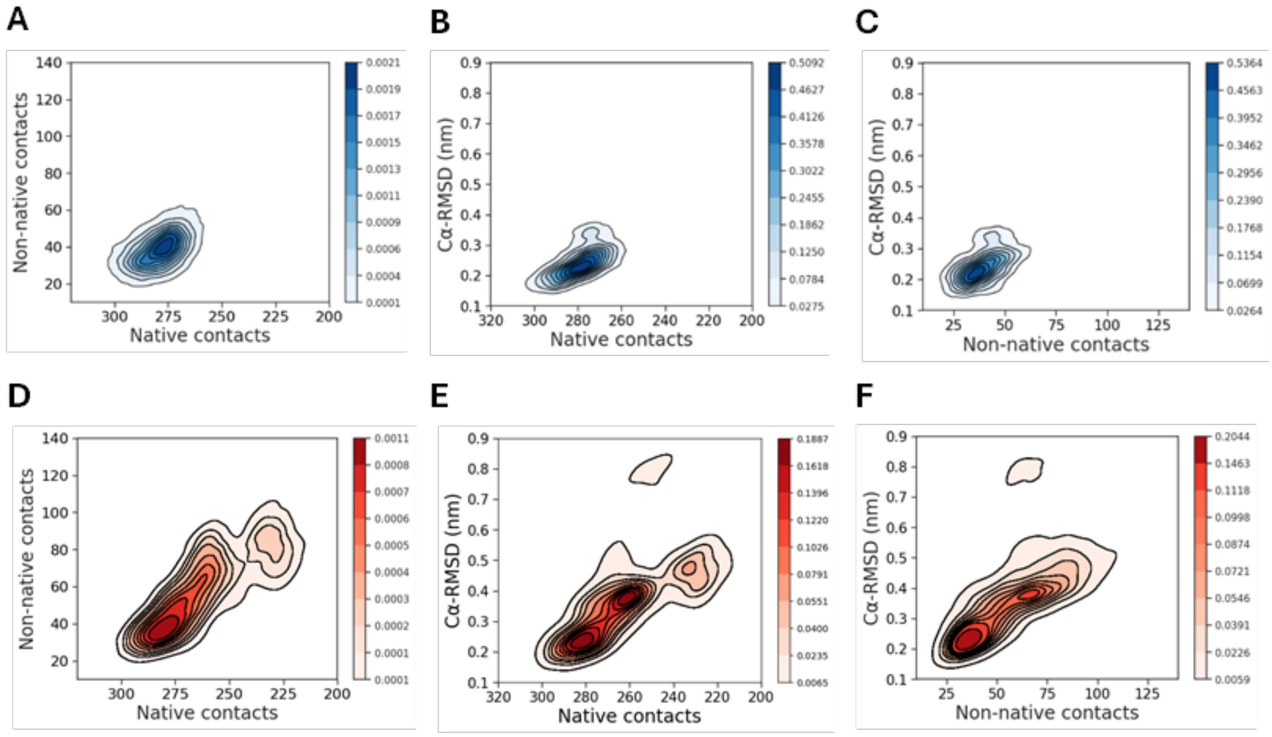

Figure S5. 2D-density distributions. WT (A-C) and T183A PrP (D-F) sampled with CS-restrained RAMD simulations were projected onto 3 coordinates, here used in pairs to generate 2D-density distributions. The three variables are (i) the number of native Cα contacts (calculated using a cutoff of 8 Å), (ii) the number of new Cα contacts (calculated using a cutoff of 8 Å), (iii) Cα-RMSD from the native structure (computed on secondary structure regions). **(A, D)** 2D-distributions projected onto native and non-native Cα contacts. **(B, E)** 2D-distributions projected onto native Cα contacts and Cα-RMSD from the native structure. **(C, F)** 2D-distributions projected onto non-native Cα contacts and Cα-RMSD from the native structure.

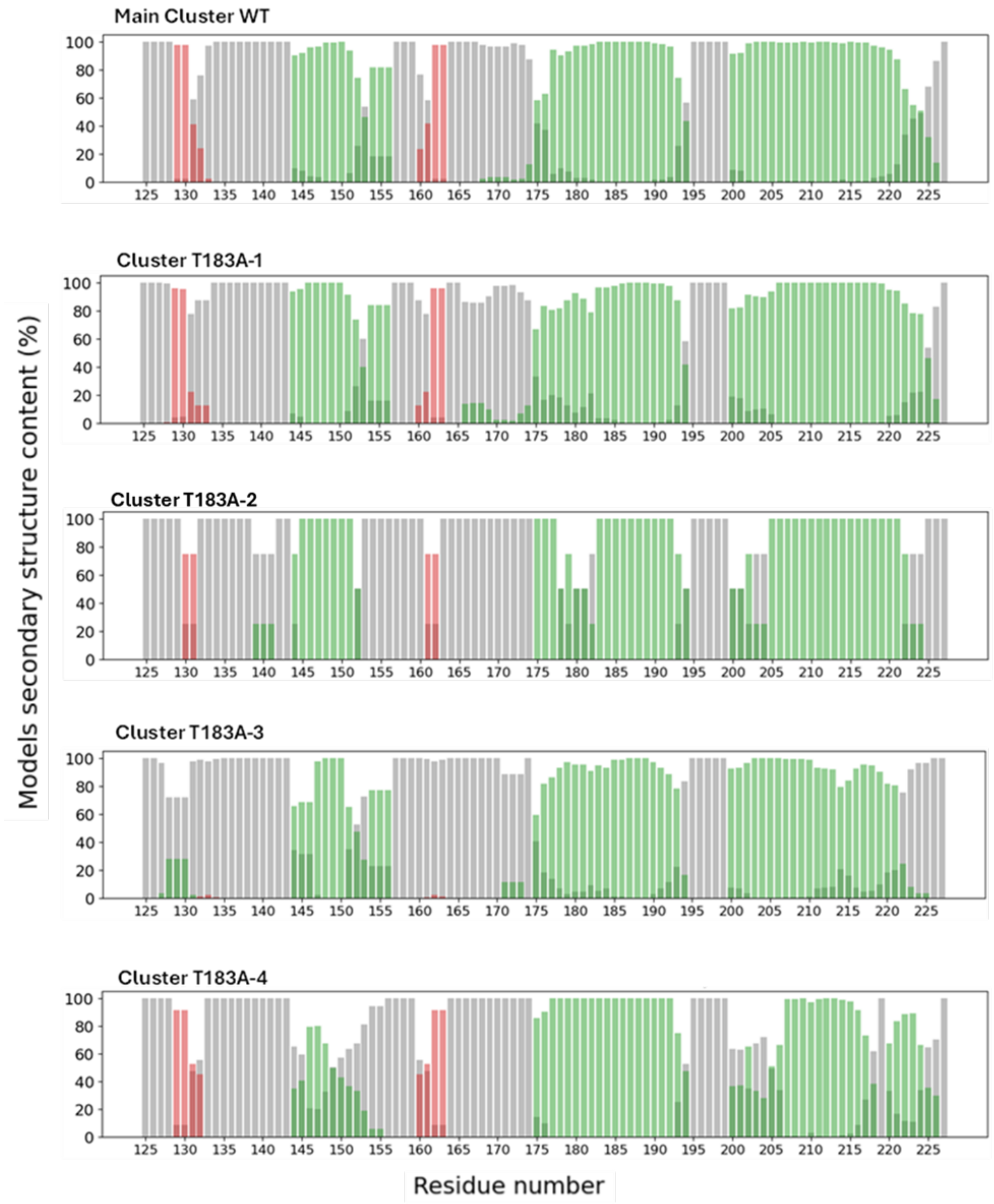

Figure S6. Secondary structure content in the conformational clusters of WT and T183A samplings. For each residue along the sequence, the percentage of secondary structure elements assigned by DSSP and across the models of each cluster is reported. Populations of α-helices, β-strands, and coil regions are shown in green, red and grey bars, respectively.

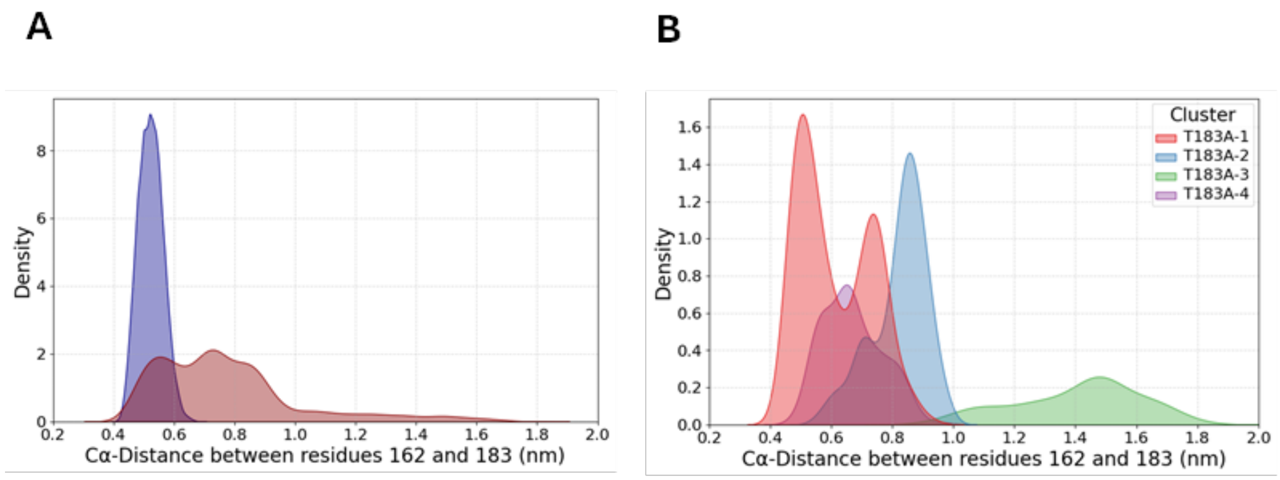

Figure S7. Native packing of strand S2 onto helix H2. The distance between Cα atoms of residues Y162 and T183/A183 has been monitored in the WT and T183A PrP ensembles. **A)** Overall distributions in the WT (blue) and T183A (red) ensemble, highlighting the disruption of the native interface between the strand S2 and the helix H2 (Figure S1) in the mutant. **B)** Density distributions across the four conformational clusters (T183A-1 to T183A-4) of the T183A ensemble.

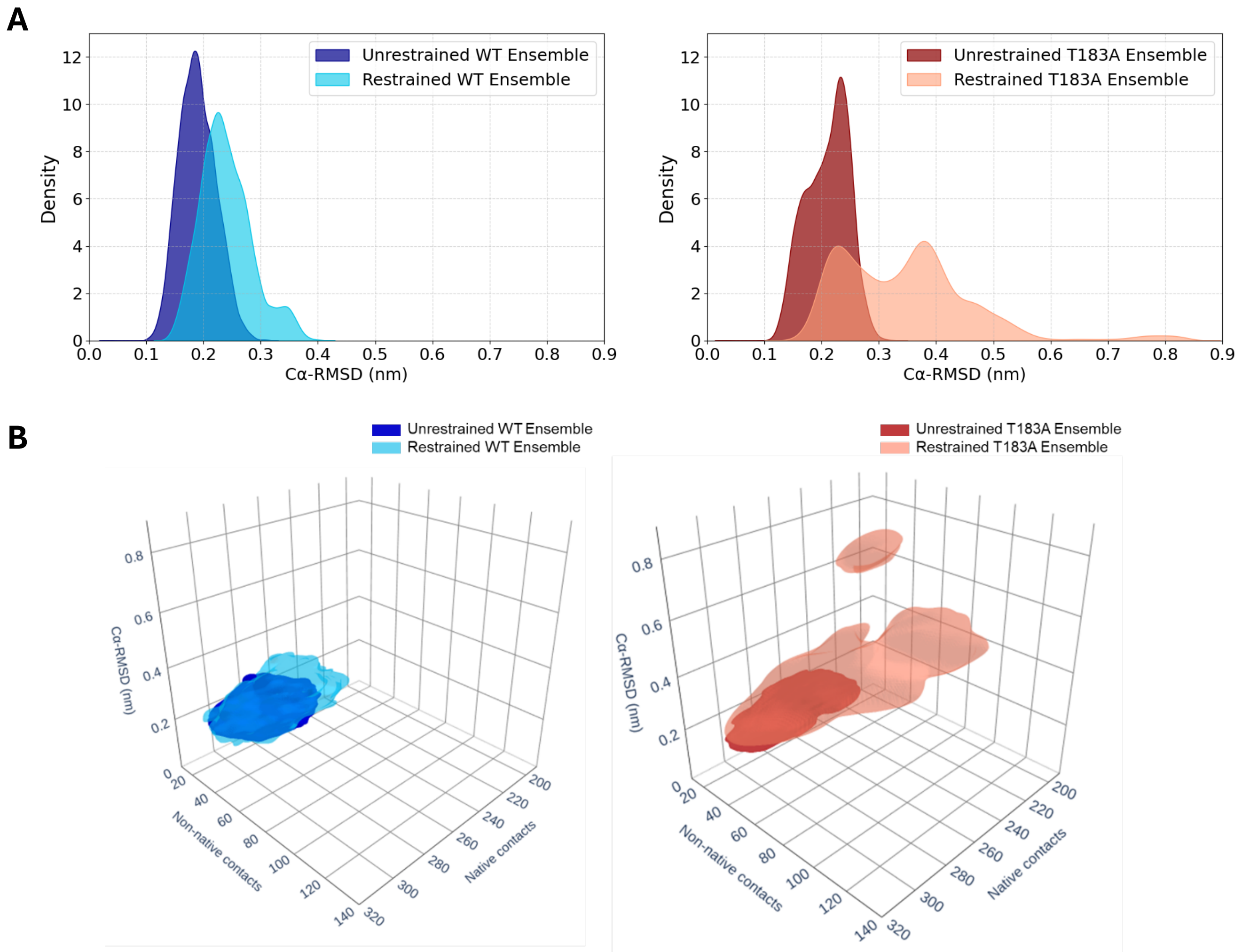

Figure S8: Unrestrained ensembles of WT and T183A $huPrP^{C}_{125\text{-}230}$ were produced using the same MD setup and by running 5 μs simulations at 310K in the absence of CS restraints. **A)** Cα-RMSD and **B)** 3D distributions show that with these simulations' setup, the unrestrained simulations mostly sample the starting free energy and are unable to describe the misfolding pathways in the case of the T183A mutant.

A **200 ps sampling phase**

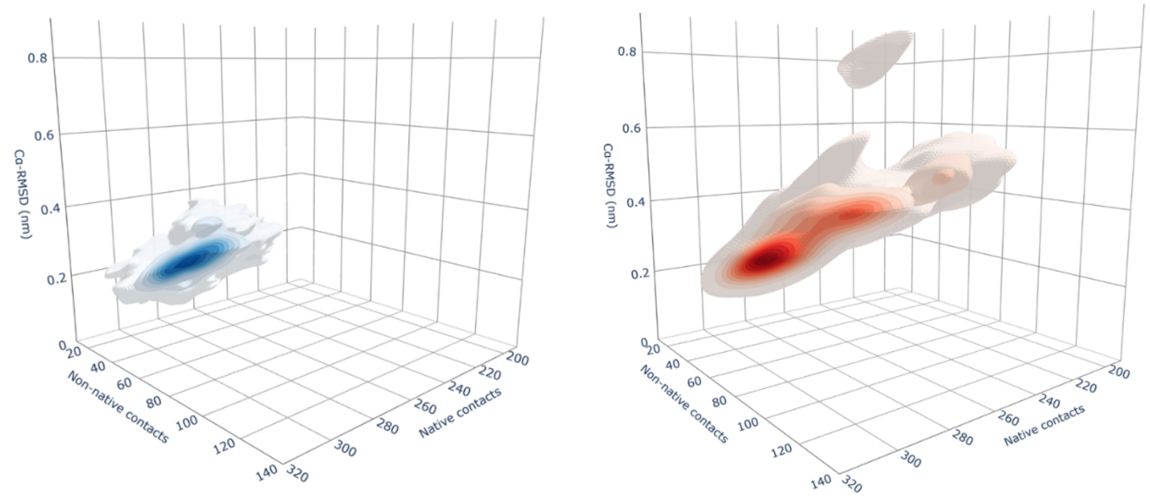

B

400 ps sampling phase

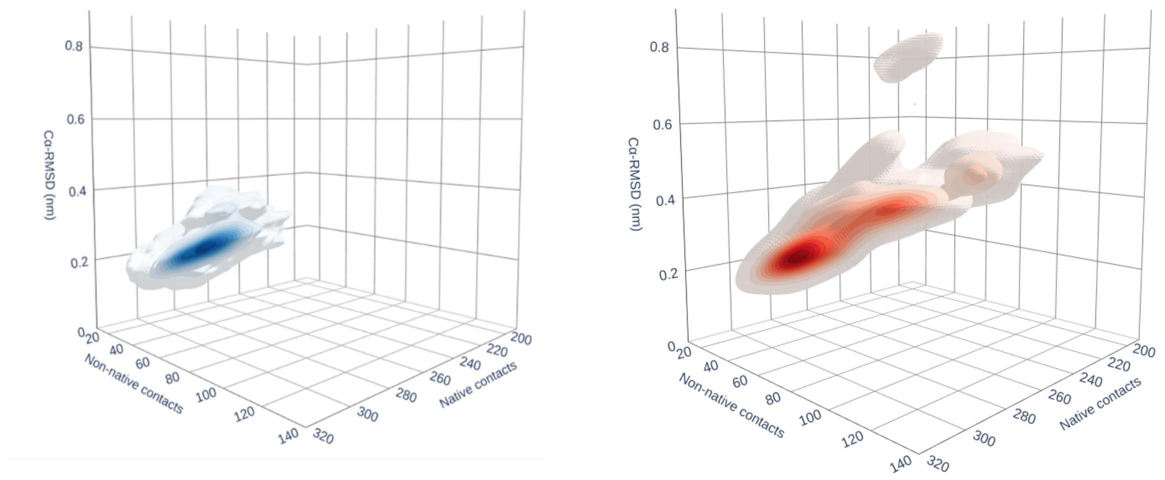

Figure S9: RAMD samplings obtained with a 400 ps sampling phase (A) overlap with those generated with a 200 ps sampling phase (B).

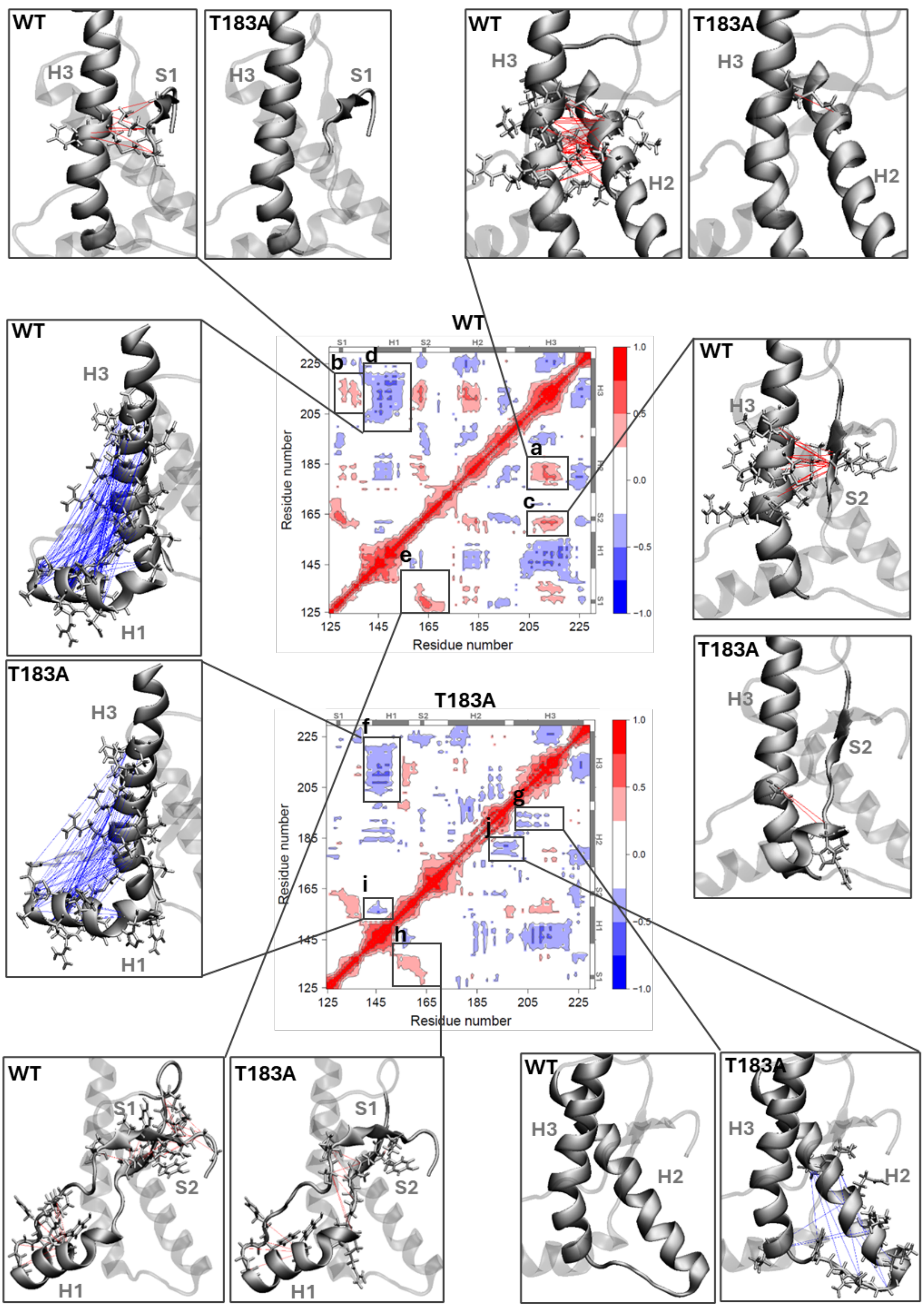

Figure S10. **Dynamic cross-correlation matrices (DCCM).** Close-up views highlighting regions with distinct correlation patterns in the of WT and T183A PrP ensembles. The central panels show full DCCM maps, with the highlighted areas indicated using black boxes. These boxes are labelled from a to j in the matrix. For each area, a close-up view shows correlated (red, correlation coefficients ≥

0.4) and anti-correlated (blue, correlation coefficients ≤ -0.4) motions between Cα atoms, plotted as connecting lines on the 3D structures of the native conformations.

References

(1) Zahn, R.; Liu, A.; Luhrs, T.; Riek, R.; von Schroetter, C.; Lopez Garcia, F.; Billeter, M.; Calzolai, L.; Wider, G.; Wuthrich, K. NMR solution structure of the human prion protein. *Proc Natl Acad Sci U S A* **2000**, *97* (1), 145-50.

(2) Knaus, K. J.; Morillas, M.; Swietnicki, W.; Malone, M.; Surewicz, W. K.; Yee, V. C. Crystal structure of the human prion protein reveals a mechanism for oligomerization. *Nat Struct Biol* **2001**, *8* (9), 770-4.

(3) Wiseman, F. K.; Cancellotti, E.; Piccardo, P.; Iremonger, K.; Boyle, A.; Brown, D.; Ironside, J. W.; Manson, J. C.; Diack, A. B. The glycosylation status of PrPC is a key factor in determining transmissible spongiform encephalopathy transmission between species. *J Virol* **2015**, *89* (9), 4738-47.

(4) Rudd, P. M.; Wormald, M. R.; Wing, D. R.; Prusiner, S. B.; Dwek, R. A. Prion glycoprotein: structure, dynamics, and roles for the sugars. *Biochemistry* **2001**, *40* (13), 3759-66.

(5) Shen, Y.; Bax, A. SPARTA+: a modest improvement in empirical NMR chemical shift prediction by means of an artificial neural network. *J Biomol NMR* **2010**, *48* (1), 13-22.

(6) Qi, G.; Vrettas, M. D.; Biancaniello, C.; Sanz-Hernandez, M.; Cafolla, C. T.; Morgan, J. W. R.; Wang, Y.; De Simone, A.; Wales, D. J. Enhancing Biomolecular Simulations with Hybrid Potentials Incorporating NMR Data. *J Chem Theory Comput* **2022**, *18* (12), 7733-50.
